# A Microfluidic Method for Simultaneous Assessment of Blood Viscosity and Red Blood Cell Aggregation During Continuous Syringe Delivery

**DOI:** 10.3390/s26092845

**Published:** 2026-05-02

**Authors:** Yang Jun Kang

**Affiliations:** Department of Mechanical Engineering, Chosun University, 10, Chosundae 1-gil, Dong-gu, Gwangju 61452, Republic of Korea; yjkang2011@chosun.ac.kr; Tel.: +82-62-230-7052; Fax: +82-62-230-7055

**Keywords:** blood viscosity, red blood cell aggregation, hemorheology, thermal-shock RBCs, sedimentation, microfluidics

## Abstract

**Highlights:**

**What are the main findings?**

- A microfluidic-based method enabled simultaneous quantification of blood viscosity and RBC aggregation index under continuous blood flow from a driving syringe.
- Hemorheological properties were strongly affected by experimental factors and thermal shock, which suppressed RBC aggregation and sedimentation.

**What are the implications of the main findings?**

- The method allows for the reliable evaluation of blood properties under dynamic flow conditions, including syringe on–off operation.
- The method could be regarded as useful for assessing RBC dysfunction and abnormal hemorheological responses in microfluidic platforms.

**Abstract:**

Accurate assessment of blood viscosity and red blood cell (RBC) aggregation under continuous flow is important for hemorheological analysis. However, simultaneous measurement remains challenging because both properties are influenced by flow conditions and RBC sedimentation. In this study, a microfluidic method is developed for the simultaneous measurement of blood viscosity and RBC aggregation index (AI) during continuous blood delivery from a driving syringe. The proposed device consists of a viscosity-sensing channel for viscosity measurement and aggregation-sensing channel for AI evaluation. The effects of flow rate, hematocrit, suspension medium, and syringe on–off operation are systematically investigated. Blood viscosity and AI are strongly affected by these factors, and transient flow interruption enhances RBC sedimentation in the syringe, thereby altering hemorheological properties. The proposed method is further used to evaluate thermally exposed RBCs, which reduce RBC aggregation and suppress RBC sedimentation when compared with control blood. At higher exposure temperatures and longer exposure times, blood viscosity and AI remain nearly constant over time, indicating minimal contribution of damaged RBCs to RBC sedimentation. These results demonstrate that the proposed method enables reliable simultaneous evaluation of blood viscosity and RBC aggregation and could be regarded as useful for detecting functional alterations of RBCs under continuous-flow conditions.

## 1. Introduction

Blood rheological properties are important clinical indicators because they directly affect flow resistance, microcirculatory perfusion, and oxygen delivery [1,2,3,4]. Whole-blood viscosity is a key determinant of vascular resistance, while RBC aggregation and deformability strongly influence blood flow behavior, particularly under low-shear and microvascular conditions [5]. Abnormal changes in these hemorheological properties are associated with impaired flow behavior in several disease states, and their measurement may therefore serve as a useful complementary approach for evaluating patient status [6], monitoring disease progression, and assessing therapeutic response [7,8,9].

Simultaneous assessment of blood viscosity and RBC aggregation is necessary to distinguish the biomechanical contributions of red blood cells and plasma proteins to hemorheological changes [10,11,12]. Blood viscosity reflects the bulk flow resistance of blood, whereas RBC aggregation is more directly associated with protein-mediated intercellular interactions and the surface or mechanical properties of red blood cells [13]. Accordingly, combined measurement of these two parameters enables more precise interpretation of whether the observed change arises predominantly from altered RBC biomechanics, modified plasma composition, or their combined effects.

Whereas conventional rheometers generally rely on larger sample volumes and bulk measurements, microfluidic chips enable physiologically relevant, low-volume, and highly integrated analysis of blood flow in microscale environments [14,15]. Several rheological properties, including blood viscosity [16,17,18,19,20,21,22,23,24,25,26], RBC aggregation [27,28,29,30,31], and RBC deformability [32,33,34,35] are assessed in a microfluidic environment.

First, microfluidic devices estimated blood viscosity indirectly by analyzing hydrodynamic responses in microscale channels, including pressure drop, blood flow rate, and coflowing interface between two fluids. The most common working principle is pressure-drop viscometry, where blood is driven through a microchannel. Blood viscosity is then obtained using a Hagen–Poiseuille equation (i.e., pressure drop = fluidic resistance × flow rate) [15,36,37]. A second approach is coflowing or interface viscometry, where blood and reference fluid are simultaneously flowed in a single channel [20]. Blood viscosity is evaluated by assessing the interface position between two streams. More recent methods determine blood viscosity by tracking blood flow during capillary filling [16,24] or pulsating motion in microchannels, which enables measurement of viscosity changes over time.

Second, RBC aggregation is typically measured by controlling the local shear condition and quantifying the optical signal generated by red blood cell clustering [38,39,40]. Because RBCs tend to form aggregates at low shear rates, most previous methods require temporary interruption of blood flow during measurement. During stasis, RBC aggregates form, and their aggregation index is calculated from timelapse image intensity [41], transmitted/backscattered light intensity [30,39,42], and electrical signal [43,44] within a specific region of interest. Some devices also use paired channel sections with different widths, so RBCs aggregate in a wide low-shear channel and are broken apart in a narrow high-shear channel [45]. The degree of aggregation is then estimated from the optical difference or intensity ratio between the aggregated and disaggregated states [46].

Although previous microfluidic methods have demonstrated on-chip measurement of blood viscosity and RBC aggregation [41,45,47,48], many of them still rely on interrupted flow, stationary-flow phases, or programmed stepwise flow control for assessing RBC aggregation effectively. To improve a real-time hemorheological monitoring, it is necessary to adopt simultaneous assessment of blood viscosity and RBC aggregation under continuous blood flow without intentional interruption of the blood stream. More recently, to resolve the issue, our group suggested a new method for probing RBC aggregation in continuous fashion [49]. However, the method shows a limitation on simultaneous measurement of blood viscosity.

In this study, a new method is proposed to simultaneously measure blood viscosity and RBC aggregation under continuous blood flow without intentional flow interruption. For this purpose, a microfluidic chip is newly designed to achieve the goal. Blood and a reference fluid (1× PBS) are delivered into the microfluidic chip with two syringe pumps. Based on our previous method [49], for inducing RBC aggregation under continuous-flow conditions, a bifurcation channel is branched from the main channel. The main channel provides a high shear-rate region, whereas the bifurcation channel provides a low shear-rate region that promotes RBC aggregation. The RBC aggregation index (AI) is then determined by analyzing image intensity values within the branched channel and main channel. In addition, based on the coflowing stream method, blood viscosity is obtained from interfacial location between two fluids.

As summarized in Table 1, many previous methods have reported simultaneous measurement of blood viscosity and RBC aggregation. However, those methods rely on precise control of the blood flow rate (i.e., periodic on–off, stepwise varying flow rate, and low shear flow), whereas the present method enables simultaneous assessment during continuous blood delivery from a driving syringe. The present method has several advantages over previous approaches. It allows for the simultaneous quantification of blood viscosity and RBC aggregation in a single microfluidic system, reduces the need for individual measurements, and enables real-time evaluation under continuous syringe-driven flow. In addition, it can reflect dynamic effects such as RBC sedimentation and flow interruption, which are not easily captured by conventional methods. The method also provides a simple image-based platform for quantitative hemorheological analysis and is sufficiently sensitive to detect functional alterations in thermally damaged RBCs.

## 2. Materials and Methods

### 2.1. Microfluidic Chip and Experimental Setup

**Figure 1:**
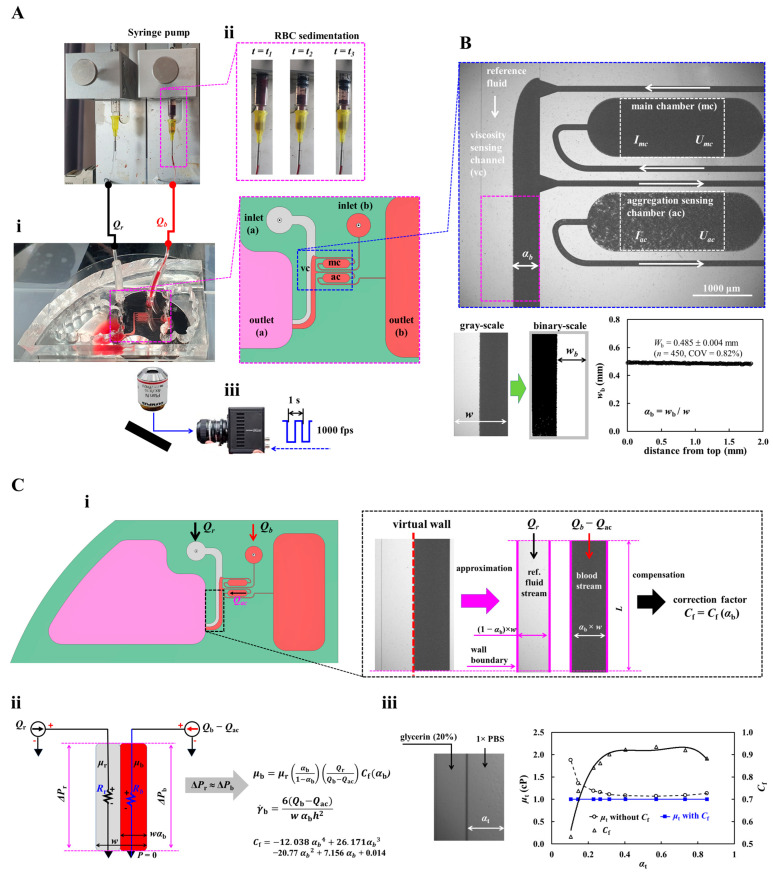
A microfluidic platform proposed for assessing blood viscosity and RBC aggregation in continuous blood flow. (**A**) Experimental setup, including a microfluidic device, two syringe pumps, and image acquisition system. (**i**) Microfluidic device was designed to have two inlets (a, b); main channel (mc), viscosity-sensing channel (vc), and aggregation-sensing channel (ac); and two outlets (a, b). (**ii**) Two syringe pumps for supplying blood and reference fluid (1× PBS). Flow rate of each fluid was set to *Q_b_* (blood) and *Q_r_* (1× PBS). Right-side panel shows RBC sedimentation in a driving syringe during blood delivery. (**iii**) Image acquisition system, including a microscope (4× objective lens, NA = 0.1), high-speed camera (1000 fps), and function generator (triggering period: 1 s). (**B**) Quantification of blood velocity (*U_mc_*, *U_ac_*), imaging intensity (*I_mc_*, *I_ac_*), and interface (*α_b_*) in the microfluidic channels. Herein, the ROI of the main channel (mc) and aggregation-sensing channel (ac) was selected within the large chamber. The ROI of the viscosity-sensing channel (vc) was positioned slightly below the junction point of the aggregation channel. The ROI of each channel was set to 1.8 mm^2^. (**C**) Blood viscosity measurement using the coflowing streams method. (**i**) Flow rate of each stream (blood stream: *Q*_b_ – *Q*_ac_; reference stream: *Q*_r_) in the ROI of the viscosity-sensing channel. Based on the virtual wall concept, coflowing streams were divided into two isolated channels. A specific correction factor was introduced to compensate for the difference between real physics and mathematical approximation. (**ii**) Viscosity formula using coflowing streams method. (**iii**) Correction factor (*C*_f_) for accurate viscosity measurement [64].

To measure blood viscosity and RBC aggregation under continuous blood flow, as shown in Figure 1A, an experimental setup was composed of a microfluidic chip, two syringe pumps, and image acquisition system.

As depicted in Figure 1(Ai), a microfluidic chip was designed to have two inlets (a, b); a main channel (mc), viscosity-sensing channel (vc), and aggregation-sensing channel (ac); and two outlets (a, b). Compared with our previous study [49], to measure blood viscosity, the main channel was connected to the viscosity-sensing channel. Blood was loaded through the inlet port of the main chamber. Starting from inlet (b), there was a narrow-sized channel (width = 0.1 mm, length = 4.9 mm), the main chamber (width = 1 mm, length = 3 mm), and another narrow channel (width = 0.1 mm and length = 8.8 mm), which connects to the large channel (width = 1 mm, length = 14.5 mm). The main chamber and aggregation-sensing chamber had the same dimensions. Starting from the inlet (a), the large channel was connected with chambers mc and ac and flowed into the outlet (a). All channels had a uniform depth of *h* = 0.05 mm.

A four-inch silicon master mold was fabricated using standard MEMS processes, including photolithography and deep reactive ion etching [53,54]. PDMS (Sylgard 184, Dow Corning, Midland, MI, USA) was prepared by mixing the elastomer base and curing agent at a 10:1 ratio (*w*/*w*), degassing the mixture under vacuum for 1 h, and curing it at 65 °C for 2 h. After curing, the PDMS was peeled from the master, trimmed, and punched to form two inlets (outer diameter = 2 mm) and two outlets. It was then bonded to a glass substrate by oxygen plasma treatment (CUTE-MPR, Femto Science Co., Ltd., Hwaseong-si, Republic of Korea) and heated at 120 °C for 10 min to strengthen PDMS–glass adhesion.

Each syringe was equipped with a 20-gauge needle and filled with blood (0.3 mL) or 1× PBS (1 mL). The needle was linked to the inlet port using polyethylene tubing (i.d. 0.25 mm, length 300 mm). Before the experiment, the microchannels were coated with 0.2% BSA for 10 min to prevent nonspecific protein adsorption, followed by washing with 1× PBS.

As shown in Figure 1(Aii), test blood and reference fluid were supplied through separate inlets of a microfluidic chip. Herein, 1× PBS was selected as reference fluid. The corresponding flow rate of each fluid was denoted as *Q_b_* (test blood) and *Q_r_* (reference fluid), respectively. During blood delivery, RBC sedimentation proceeded continuously depending on RBC mechanical properties [55], hematocrit [56,57], and blood medium [29,58,59]. Continuous RBC sedimentation contributed to changes in hematocrit of test blood flowing in the microfluidic chip [60,61]. The right-side panel represents a timelapse snapshot for RBC sedimentation over time.

The device was observed under an inverted microscope (IX81, Olympus, Tokyo, Japan) fitted with a 4× objective (NA = 0.10). Flow images were captured at 1000 frames/s using a high-speed camera with an external trigger every 1 s, and all tests were carried out at room temperature (25 °C).

### 2.2. Quantification of Blood Velocity, Image Intensity, and Interface

**Figure A1:**
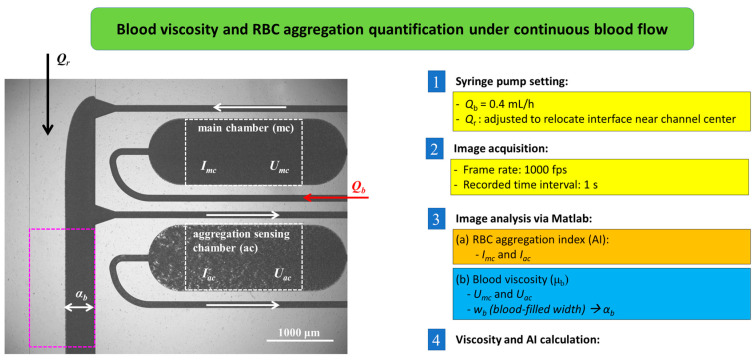
Workflow of measurement procedures such as syringe pump setting, image acquisition, image analysis, and viscosity and AI calculation. The arrow (‘→’) denoted flow direction of each fluid in microfluidic channels.

In this study, blood velocity in the main and aggregation channels was measured simultaneously to determine the flow rate entering the viscosity-sensing channel from the syringe. As shown in Figure A1 (Appendix A), RBC aggregation index was obtained from image intensity in the main and aggregation-sensing channels. Furthermore, blood viscosity was estimated from the interface in the viscosity-sensing channel.

First, for quantifying average blood velocity in the main and aggregation-sensing channels, a specific ROI (1.8 mm^2^) was selected in the largest channel region. Timelapse velocity fields were analyzed using PIVlab (Version: 3.12) [62] with an interrogation window of 67 × 67 µm^2^ and 50% overlap, and the resulting vectors were filtered using local median and standard-deviation methods. Since the DOC was estimated to be greater than 300 µm and exceeded the channel depth of 50 µm [63], the measured velocities were assumed to represent depth-averaged velocity. Average velocities over each ROI were then defined as *<U_mc_>* and *<U_ac_>* and were used to calculate *Q_ac_ =* (<*U_ac_>/<U_mc_*>) *× Q_b_* and *Q_vc_ = Q_b_ − Q_ac_.*

Second, to quantify RBC aggregation, each image was subtracted from the initial background image and analyzed using MATLAB (Version 2025b, MathWorks, Natick, MA, USA). An ROI (1.8 mm^2^) was defined in the largest region of the main and aggregation-sensing channels, and the mean grayscale intensities were obtained as *I_mc_* and *I_ac_*, respectively. The same process was applied to all image sets. The RBC aggregation index (AI) was then calculated as AI = (*I*_mc_ − *I*_ac_)/*I*_mc_ over time.

Third, to quantify the interface in the viscosity-sensing channel, as shown in the lower panel of Figure 1B, grayscale images were binarized using Otsu’s method. A 1.8 mm^2^ ROI was selected in the straight coflowing region located more than 1 mm downstream of the aggregation-sensing channel junction. White and black indicate blood and reference fluid, respectively. The interface was identified in the vertical direction. Blood-filled width (*w*_b_) was then obtained as *w*_b_ = 0.485 ± 0.004 mm (*n* = 450, and COV = 0.82%). The average blood-filled width within the ROI was defined as *w_b_*, and the normalized interface was expressed as α_b_ = *w_b_*/*w*, where channel width was denoted as *w* = 1 mm.

### 2.3. Blood Viscosity Measurement Using Coflowing Stream Method

As shown in Figure 1C, a coflowing streams method was used to obtain blood viscosity [64,65]. As shown in Figure 1(Ci), using two syringe pumps, the flow rate of test blood and 1× PBS was set to *Q_r_* and *Q_b_*, respectively. A portion of the syringe-delivered blood entered the aggregation-sensing channel at a flow rate of *Q_ac_*, while the remaining blood flowed into the viscosity-sensing channel at *Q_b_* − *Q_ac_*. In the viscosity-sensing channel, the test blood and 1× PBS flowed in parallel at *Q_b_* − *Q_ac_* and *Q_r_*, respectively.

To derive viscosity formula, as shown in right-side panel of Figure 1(Ci), based on the virtual wall concept, coflowing streams were treated as two isolated channels. That is, each channel was filled with reference fluid and blood, respectively. The equivalent fluidic resistance of each channel was established under the assumptions of incompressible, laminar, and sufficiently developed flow in channels of fixed geometry, while gravitational, entrance, and minor three-dimensional interface effects were neglected. A specific correction factor, *C*_f_, was introduced to compensate for modeling error caused by mathematical simplification. It was determined as a function of interface and channel dimension (i.e., width and depth) [47,52]. As shown in Figure 1(Cii), a simple fluidic circuit model of the coflowing stream method was constructed using a discrete fluid element, including flow rate (Q_r_, *Q_b_* − *Q_ac_*) and fluidic resistance (*R_r_*, *R_b_*). The *R_r_* and *R_b_* represent equivalent fluidic resistance of 1× PBS stream and test blood stream, respectively. Considering that both streams had the same pressure drop (i.e., Δ*P_r_* = Δ*P_b_*), the blood viscosity formula was derived as $\mu_{b}=\mu_{r}\left(\frac{\alpha_{b}}{1-\alpha_{b}}\right)(\frac{Q_{r}}{Q_{b}-{\;Q}_{ac}})C_{f}(\alpha_{b})$. As shown in Figure 1(Ciii), according to the previous study [64], the same channel dimension as the present study (i.e., width = 1 mm, and depth = 0.05 mm) was used to obtain the correction factor. In that calibration, glycerin (20%) and 1× PBS were used as reference fluid and test fluid, respectively. The interfacial position was varied by adjusting the flow rate ratio between the two fluids. Based on the viscosity formula, the correction factor was then identified as *C_f_* (*α_b_*) = −12.038 *α_b_*^4^ + 26.171 *α_b_*^3^ − 20.770 *α_b_* ^2^ + 7.156 *α_b_* + 0.014 (R^2^ = 0.970). The results showed that the correction factor improved measurement accuracy substantially [64]. In addition, the shear rate of test blood stream in the viscosity-sensing channel was derived as $\dot{\gamma }=\frac{6(Q_{b\;}-\;Q_{ac})}{\alpha_{b}w\;h^{2}}$.

### 2.4. Blood Sample Preparation

Packed RBCs were provided by the Gwangju–Chonnam Blood Bank (Gwangju, Republic of Korea) and kept refrigerated before sample preparation. Normal RBCs were isolated according to an established washing procedure [66] by sequential removal of blood medium and buffy coat, and the procedure was repeated twice. In this study, a single blood donation was used to demonstrate the proposed method.

Three sets of test blood were prepared. First, hematocrit-dependent effects were examined using normal RBCs suspended into dextran solution (20 mg/mL) at Hct = 30–60%. Second, medium-dependent effects were evaluated by suspending normal RBCs into dextran solutions of 5~20 mg/mL into 1× PBS at Hct = 50%. Third, thermally treated RBCs were prepared by incubating normal RBCs at 40~50 °C for up to 180 min, followed by washing and resuspension in dextran solution (20 mg/mL) at Hct = 50%.

## 3. Results and Discussion

### 3.1. Validation of Fluid Velocity Obtained by Micro-PIV Technique

In this subsection, because accurate viscosity measurement depends on fluid velocity, it was necessary to validate fluid velocity obtained by conducting micro-PIV technique [62]. Considering that medium and hematocrit affected blood velocity substantially [67], glycerin solution and suspended blood were used to probe measurement accuracy.

**Figure 2:**
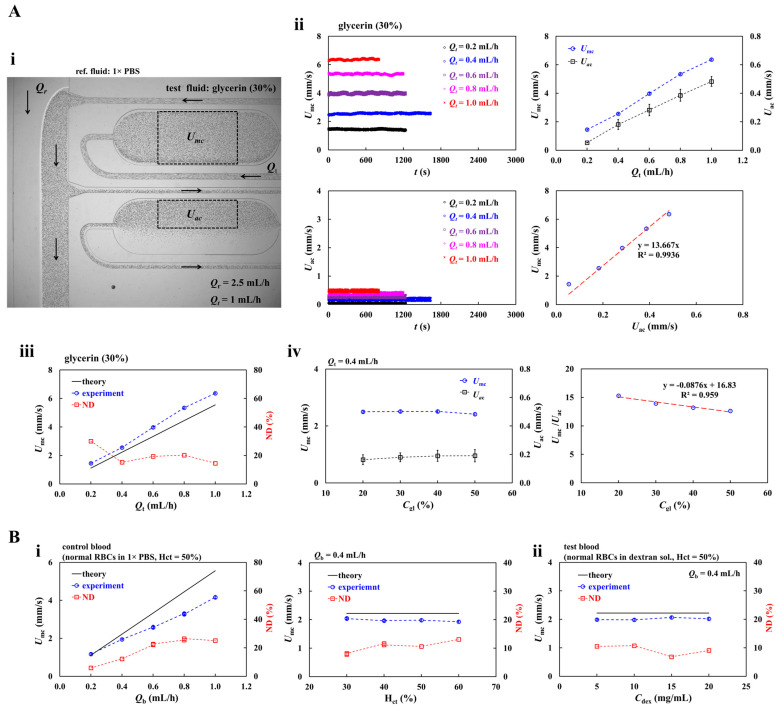
Validation of fluid velocity obtained by conducting micro-PIV technique. (**A**) Velocity measurement of glycerin solution. (**i**) Microscopic image for showing two fluids (i.e., 1× PBS: reference fluid, glycerin [30%]: test fluid) flowing in the channels. Flow rate of each fluid set to *Q*_r_ = 2.5 mL/h and *Q*_t_ = 1 mL/h, respectively. The arrow (‘→’) denotes flow direction in the channel. (**ii**) Variations in *U*_mc_ and *U*_ac_ with respect to *Q*_t_. According to linear regression, the *U*_mc_ was proportional to the *U*_ac_ (i.e., *U*_mc_ = 13.667 *U*_ac_, R^2^ = 0.9936). (**iii**) Quantitative comparison of *U_mc_* obtained by experimental results and theoretical calculation with respect to *Q*_t_. Herein, ND denotes normalized difference between theoretical calculation and experimental measurement. (**iv**) Contribution of glycerin concentration to the variations in *U*_mc_, *U*_ac_, and *U*_mc_/*U*_ac_. Linear regression analysis confirmed that *U*_mc_/*U*_ac_ was decreased linearly with respect to glycerin concentration (*C*_gl_). (**B**) Measurement of blood velocity using micro-PIV technique. Herein, hematocrit level of blood was adjusted by adding normal RBCs into medium (i.e., 1× PBS, dextran solution). (**i**) Contribution of hematocrit to *U*_mc_. The left-side panel showed variations in *U_mc_* with respect to *Q*_b_. The right-side panel exhibited variations in U_mc_ with respect to Hct, at a fixed blood flow rate of *Q*_b_ = 0.4 mL/h. (**ii**) The effect of blood medium on *U*_mc_.

First, instead of suspended blood, pure liquid (i.e., glycerin) was selected as test fluid. The concentration of glycerin solution was set to *C*_gl_ = 20%~50%. The flow rate of test fluid was set to *Q_t_* = 0.2~1.0 mL/h. As shown in Figure 2(Ai), two fluids (i.e., 1× PBS: reference fluid, glycerin [30%]: test fluid) were co-infused into each inlet port. Herein, to visualize fluid velocity of test fluid, normal RBCs were added into test fluid (i.e., test fluid: 1 mL, RBCs: 30 μL). The flow rate of each fluid was set to *Q*_r_ = 2.5 mL/h and *Q*_t_ = 1 mL/h, respectively. The arrow (‘→’) denotes flow direction in the channel. As most RBCs flowed through the upper regions of the aggregation channel, the ROI size of aggregation channel was reduced to 0.9 mm^2^. With respect to glycerin solution (30%), as shown in the left-side panel of Figure 2(Aii), timelapse *Q*_mc_ and *Q*_ac_ were obtained with respect to *Q*_t_. Both velocities remained stable over time and increased with respect to *Q*_t_. The right-side panel shows variations in *U*_mc_ and *U*_ac_ with respect to *Q*_t_. *U*_mc_ and *U*_ac_ increased linearly with respect to *Q*_t_. Additionally, according to linear regression analysis, the *U*_mc_ was proportional to the *U*_ac_ (i.e., *U*_mc_ = 13.667 *U*_ac_, R^2^ = 0.9936). These results indicated that 7.3% of the test fluid entered the aggregation channel, while the remaining 92.7% flowed into the viscosity-sensing channel. To compare quantitatively, as depicted in Figure 2(Aiii), measured and calculated *U*_mc_ values were plotted as a function of *Q*_t_. Except for *Q*_t_ = 0.2 mL/h, the normalized difference (ND) was estimated to be approximately 20%. The results indicated that the micro-PIV technique overestimated the velocity of the glycerin solution by about 20%. As shown in Figure 2(Aiv), the time-courses of *U*_mc_ and *U*_ac_ were measured over glycerin concentration ranges of *C*_gl_ = 20%~50%. Both velocities remained stable over the range of glycerin concentration. According to linear regression analysis, *U*_mc_/*U*_ac_ decreased linearly with respect to *C*_gl_ (i.e., *U*_mc_/*U*_ac_ = −0.0876 *C*_gl_ + 16.83, R^2^ = 0.959). The results indicated that *U*_mc_/*U*_ac_ depended on glycerin concentration.

Second, blood velocity was evaluated by varying hematocrit and suspending medium (1× PBS or dextran solution). As shown in the left-side panel of Figure 2(Bi), control blood was prepared by suspending normal RBCs into 1× PBS. The measured and calculated *U*_mc_ values were plotted along *Q*_b_. The normalized difference (ND) increased markedly up to *Q*_b_ = 0.6 mL/h and then reached a plateau above this flow rate. These results indicate that micro-PIV underestimated blood velocity by less than 25%.

As shown in the right-side panel of Figure 2(Bi), *U*_mc_ was also examined with respect to hematocrit at a fixed flow rate of *Q*_b_ = 0.4 mL/h. From the results, the ND remained below 13.1%. In addition, as shown in Figure 2(Bii), to assess the effect of the suspending medium on velocity measurement, test blood (Hct = 50%) was prepared by suspending normal RBCs into dextran solution (*C*_dex_ = 5~20 mg/mL). The ND was below 11% over this concentration range.

**Figure 3:**
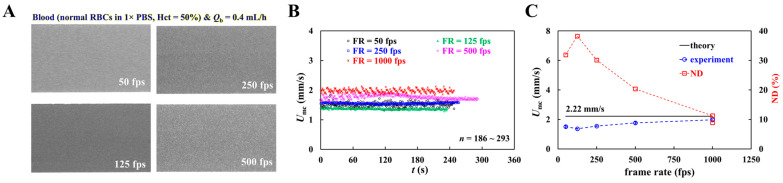
Contribution of frame rate to fluid velocity obtained by micro-PIV technique. (**A**) Variations in microscopic image with respect to frame rate (FR) (i.e., FR = 50, 125, 250 and 500 fps). (**B**) Time-course of *U_mc_* with respect to frame rate (FR). (**C**) Measured and calculated *U*_mc_ values with respect to frame rate. The *U*_mc_ obtained at each frame rate is summarized as mean ± standard deviation.

Finally, the effect of the camera frame rate on the fluid-velocity measurement was evaluated by varying the frame rate from 50 fps to 1000 fps. Blood (Hct = 50%) suspended in 1× PBS was delivered through the main channel at a constant flow rate of 0.4 mL/h. Figure 3A is presented representative microscopic images obtained at different frame rates. Figure 3B summarizes the time-course of *U_mc_* as mean ± standard deviation. As shown in Figure 3C, the discrepancy between measured and calculated *U_mc_* decreased markedly with increasing frame rate. Based on these results, a frame rate of 1000 fps was adopted for subsequent experiments.

Experimental results led to the conclusion that the micro-PIV technique could effectively track the time-course of fluid velocity. However, as systematic underestimation of approximately 20–25% in the raw micro-PIV velocity measurements was observed, calibration was required to obtain an accurate flow rate of test blood. In the following experiments, the flow rates in the test and aggregation channels (*Q*_mc_, *Q*_ac_) were calibrated from measured blood velocity using the micro-PIV technique.

### 3.2. Demonstration of the Proposed Method

To demonstrate the proposed method, control blood and test blood were prepared by suspending normal RBCs into 1× PBS and dextran solution (20 mg/mL), respectively. The hematocrit of each blood was set to Hct = 50%. Herein, the blood flow rate was set to *Q_b_* = 0.4 mL/h. Based on the shear-rate formula of a low-aspect rectangular channel (i.e., $\dot{\gamma }=\frac{6\;Q}{w\;h^{2}}$) [37], the corresponding shear rate of wide-width channel and narrow-width channel was estimated as $\dot{\gamma }=\;$266.7 s^−1^ (wide-width channel) and $\dot{\gamma }=\;$2666.7 s^−1^ (narrow-width channel), respectively.

**Figure 4:**
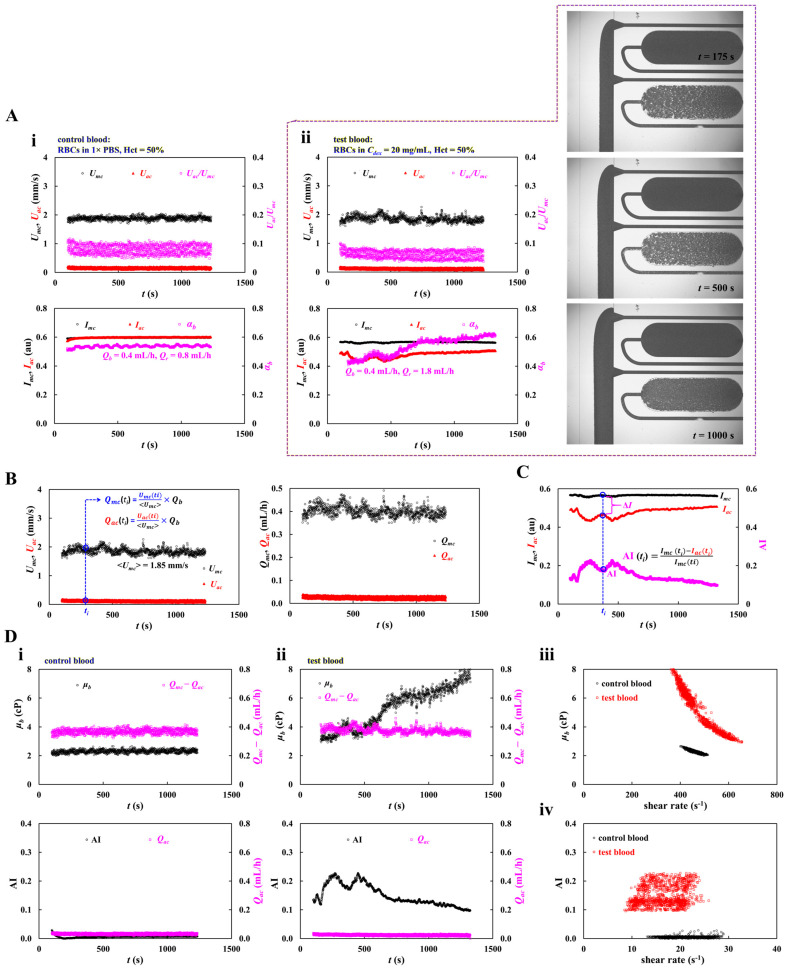
Quantitative comparison between control blood and test blood using blood viscosity and aggregation index in continuous blood flow. (**A**) Preliminary demonstration of the proposed method. Herein, hematocrit of two bloods (control blood and test blood) were adjusted to Hct = 50% by suspending normal RBCs into 1× PBS and dextran solution (20 mg/mL), respectively. (**i**) Timelapse blood velocity (*U_mc_*, *U_ac_*), image intensity (*I_mc_*, *I_ac_*), and interface (α_b_) of control blood. Herein, flow rate of each fluid was set to *Q_b_* = 0.4 mL/h and *Q_r_* = 0.8 mL/h, respectively. (**ii**) Timelapse blood velocity (*U_mc_*, *U_ac_*), image intensity (*I_mc_*, *I_ac_*), and interface (α_b_) of test blood. Herein, flow rate of each fluid was set to *Q_b_* = 0.4 mL/h and *Q_r_* = 1.8 mL/h, respectively. Right-side panel depicts microscopic images captured at *t* = 175, 500, and 1000 s. (**B**) Calibration of *Q*_mc_ and *Q*_ac_ using steady plateau value of *U*_mc_. Left-side panel represents timelapse *U*_mc_ and *U*_ac_. Herein, the steady plateau value of *U*_mc_ was obtained as <*U*_mc_> = 1.85 mm/s. The corresponding flow rate of each velocity was calibrated as *Q*_mc_ = *U*_mc_/<*U*_mc_> × *Q*_b_ and *Q*_ac_ = *U*_ac_/<*U*_mc_> × *Q*_b_, respectively. Right-side panel shows timelapse *Q*_mc_ and *Q*_ac_. (**C**) Aggregation index (AI) calculation using *I_mc_* and *I_ac_*. As the ΔI (i.e., ΔI = *I_mc_* − *I_ac_*) was proportional to RBC aggregation; the AI as normalized form is defined as AI = (*I_mc_* − *I_ac_*)/*I_mc_*. (**D**) Quantitative comparison between control blood and test blood. (**i**) Timelapse *μ_b_* and AI of control blood. (**ii**) Timelapse *μ_b_* and AI of test blood. (**iii**) Comparison of blood viscosity between two bloods. (**iv**) Comparison of AI between two bloods.

First, as shown in Figure 4(Ai), with respect to control blood, timelapse blood velocity (*U_mc_*, *U_ac_*), image intensity (*I_mc_*, *I_ac_*), and interface (*α_b_*) were obtained. Herein, to relocate the interface near the channel center position, flow rate of 1× PBS was set to *Q_r_* = 0.8 mL/h. All parameters remained constant over the period. On the other hand, as depicted in Figure 4(Aii), with regard to test blood, timelapse blood velocity (*U_mc_*, *U_ac_*), image intensity (*I_mc_*, *I_ac_*), and interface (*α_b_*) were obtained. Initially, to relocate the interface near the channel center, the flow rate of 1× PBS was set to *Q_r_* = 1.8 mL/h. The right-side panel depicts microscopic images captured at *t* = 175, 500, and 1000 s. When compared with the main channel and viscosity-sensing channel, RBC aggregation was clearly observed within aggregation-sensing channel. *U_mc_*, *U_ac_*, and *U_ac_*/*U_mc_* did not show substantial variations over time, whereas the blood flow rate maintained constant. However, as shown in Figure 1(Aii), dextran solution (20 mg/mL) caused RBC sedimentation in a driving syringe during blood delivery. Accordingly, a clear difference in image intensity (i.e., Δ*I* = *I_mc_* − *I_ac_*) was observed, which gradually decreased over time. The interface increased progressively. From the results, image intensity and interface could be used to detect substantial differences between blood samples.

Second, the blood velocity had to be calibrated to the obtain flow rate. Because micro-PIV measurements were affected by hematocrit [67], velocity alone was insufficient for accurate flow rate estimation. As the test blood was delivered at a constant flow rate, the measured velocity could be used to determine flow rate after calibration. As shown in the left-side panel of Figure 4B, timelapses *U_mc_* and *U_ac_* of the test blood were plotted to explain the calibration procedure. The steady plateau value of *U_mc_* was obtained as <*U_mc_*> = 1.85 mm/s. The corresponding flow rates calculated from each velocity were calibrated as *Q_mc_* = *U_mc_*/<*U_mc_*> × *Q_b_* and *Q_ac_* = *U_ac_*/<*U_mc_*> × *Q_b_*, respectively. The right-side panel of Figure 4B shows timelapse *Q_mc_* and *Q_ac_*. As a result, the flow rates of *Q_mc_* and *Q_ac_* were obtained as *Q_mc_* = 0.40 ± 0.02 mL/h (*n* = 1224) and *Q_ac_* = 0.02 ± 0.01 mL/h (*n* = 1224), respectively. The results indicated that only 5% of the test blood entered the aggregation channel, whereas the remaining 95% of the blood flowed into the viscosity-sensing channel.

Third, based on our previous method [49], RBC aggregation index (AI) was calculated using timelapse *I_mc_* and *I_ac_*. As shown in Figure 4A, with regard to control blood, *I_mc_* and *I_ac_* were overlapped over time. However, when 1× PBS was replaced by dextran solution (20 mg/mL), the *I_ac_* was decreased substantially when compared with *I_mc_*. That is, RBC aggregation contributed to decreasing *I_ac_* considerably. The Δ*I* (i.e., Δ*I* = *I_mc_* − *I_ac_*) represents the strength of RBC aggregation appropriately [49]. As shown in Figure 4C, by normalizing Δ*I* with *I_mc_*, the RBC aggregation index was expressed as AI = (*I_mc_* − *I_ac_*)/*I_mc_*. This simple equation enabled continuous timelapse measurement of AI without stopping the blood flow.

Finally, based on the calculation procedures for flow rate, blood viscosity, and the RBC aggregation index, as shown in Figure 4D, control and test blood samples were quantitatively compared using viscosity and the RBC aggregation index. As shown in Figure 4(Di), with regard to control blood, the upper panel exhibits timelapse *μ_b_* and *Q_mc_* − *Q_ac_*. The lower panel shows timelapse AI and *Q_ac_*. Accordingly, the *μ_b_* was obtained as *μ_b_* = 2.31 ± 0.11 cP. Herein, the number of data points is denoted as *n* = 1134. The *Q_mc_* − *Q_ac_* was acquired as *Q_mc_* − *Q_ac_* = 0.37 ± 0.02 mL/h. In addition, AI and *Q_ac_* were obtained as AI = 0.01 ± 0.003 and *Q_ac_* = 0.03 ± 0.01 mL/h. As depicted in Figure 4(Dii), timelapse *μ_b_* and AI of test blood were obtained. The upper panel exhibits timelapse *μ_b_* and *Q_mc_* − *Q_ac_*. During blood delivery, blood viscosity increased significantly from *μ_b_* = 2.93 cP to *μ_b_* = 8.15 cP. Herein, the amount of data is denoted as *n* = 1165. The *Q_mc_* − *Q_ac_* maintained constant as *Q_mc_* − *Q_ac_* = 0.38 ± 0.02 mL/h. Accordingly, RBC sedimentation in a driving syringe proceeded over time [60], which contributed to increasing blood viscosity over time. However, the flow rate of *Q_mc_* and *Q_ac_* maintained constant because flow rate of test blood set to *Q_b_* = 0.4 mL/h. The lower panel shows timelapse variations in AI and *Q_ac_*. Initially, AI was 0.13 and gradually increased to 0.23. It then fluctuated between 0.23 and 0.17 before eventually decreasing from 0.23 to 0.10 over time. In contrast, *Q_ac_* remained nearly constant at 0.025 ± 0.006 mL/h. These results indicated that the RBC aggregation index changed continuously with the progression of RBC sedimentation in the driving syringe. Shear rate in the viscosity-sensing channel was estimated using timelapse *α_b_* and (*Q_mc_* − *Q_ac_*). As shown in Figure 4(Diii), blood viscosity (*μ_b_*) of each blood sample was plotted as a function of shear rate ($\dot{\gamma }$). The viscosity of test blood was much larger than that of control blood. As RBC sedimentation in the test blood progressed over time, the interface increased substantially, which resulted in a decreasing shear rate. Figure 4(Div) shows variations in AI between the two bloods with respect to shear rate. Compared with the control blood, the test blood showed a higher AI, while its shear rate was slightly reduced as a result of *Q_ac_*.

The quantitative comparison demonstrated that the proposed method was effective for discriminating between control and test blood during continuous flow from a driving syringe.

### 3.3. Quantitative Evaluation of Glycerin Viscosity Obtained by Coflowing Method

In this subsection, several concentrations of glycerin solution were used to probe accuracy of fluid viscosity obtained by the coflowing method. Herein, the concentrations of glycerin solution were set to *C*_gl_ = 20%~50%. As shown in Figure 2(Ai), 1× PBS was used as reference fluid.

**Figure 5:**
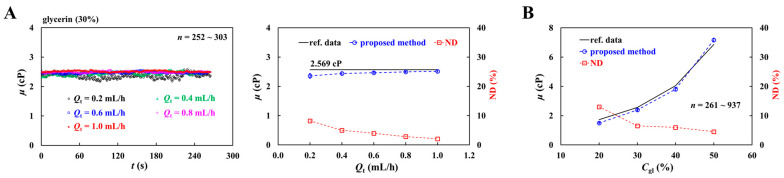
Quantitative evaluation of viscosity of glycerin solution with coflowing method. (**A**) Contribution of supplied flow rate to glycerin viscosity. Left-side panel shows time-course of glycerin viscosity with respect to *Q*_t_ = 0.2~1.0 mL/h. Herein, 30% glycerin solution was used as test fluid. Right-side panel depicts quantitative comparison between reference data and proposed method. The normalized difference (ND) is plotted along *Q*_t_. (**B**) Quantitative comparison between reference data and proposed method.

First, to validate the viscosity measurement, 30% glycerin as a Newtonian fluid was selected and supplied at flow rates of 0.2~1.0 mL/h. As shown in the left-side panel of Figure 5A, the timelapse viscosity of the glycerin solution was acquired with respect to *Q*_t_. The results indicated that glycerin viscosity remained stable over time. The right-side panel of Figure 5A shows variations in glycerin viscosity with respect to Q_t_. Compared with the reference viscosity of 2.569 cP [68], the proposed method underestimated the viscosity by less than 10%. As glycerin viscosity remained constant over the specific range of flow rate, it was confirmed that the glycerin solution behaved as a Newtonian fluid. From the results, the proposed coflowing method provided reliable and consistent viscosity measurement for Newtonian fluid.

Second, glycerin solutions with different concentrations were prepared to vary viscosity systematically. As shown in Figure 5B, the viscosities measured by the proposed method were compared with reference values as a function of glycerin concentration (*C*_gl_). The normalized difference remained below 13% over the tested concentration range of the glycerin solution.

From the experimental measurements, the proposed coflowing method was able to measure fluid viscosity with sufficient accuracy.

### 3.4. Contribution of Blood Flow Rate, Hematocrit, and Suspending Medium

According to the previous studies, blood viscosity and AI were significantly influenced by flow condition, hematocrit, and suspending medium [5,10,51,69,70,71,72]. In this section, the proposed method was used to evaluate the effects of several factors on both properties under a constant blood flow rate, with blood continuously delivered from a driving syringe into the microfluidic chip.

**Figure 6:**
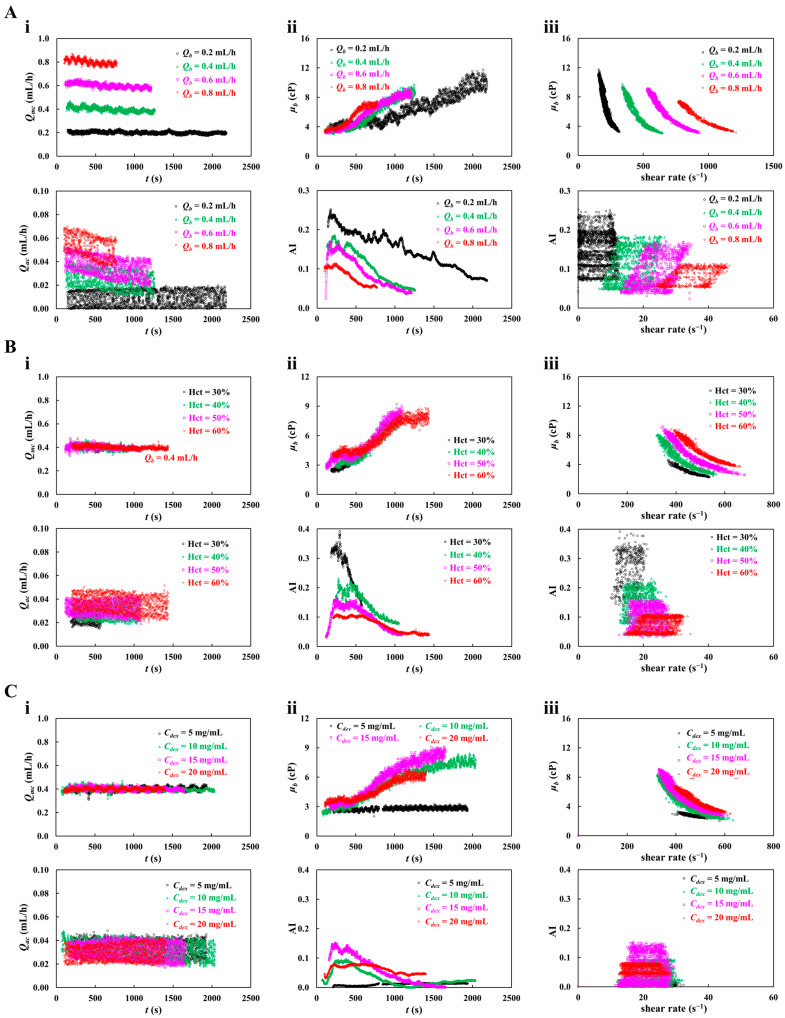
Contribution of flow rate, hematocrit, and blood medium (dextran solution) to blood viscosity and RBC aggregation. (**A**) The effect of blood flow rate on blood viscosity (*μ_b_*) and RBC aggregation index (AI). Herein, test blood (Hct = 50%) was prepared by suspending normal RBCs into dextran solution (20 mg/mL). (**i**) Timelapse *Q_mc_* and *Q_ac_* with respect to *Q_b_* = 0.2~0.8 mL/h. (**ii**) Timelapse *μ_b_* and AI with respect to *Q_b_*. (**iii**) Variations in *μ_b_* and AI as a function of shear rate ($\dot{\gamma }$) with respect to *Q_b_*. (**B**) Contribution of hematocrit (Hct) to blood viscosity (*μ_b_*) and RBC aggregation index (AI). Herein, hematocrit of test blood was adjusted to Hct = 30%~60% by adding normal RBCs into dextran solution (20 mg/mL). Flow rate of blood was fixed at *Q_b_* = 0.4 mL/h. (**i**) Timelapse *Q_mc_* and *Q_ac_* with respect to Hct. (**ii**) Timelapse *μ_b_* and AI with respect to Hct. (**iii**) Variations in *μ_b_* and AI as a function of shear rate ($\dot{\gamma }$) with respect to Hct. (**C**) Contribution of dextran solution to blood viscosity and RBC aggregation index. Herein, hematocrit and blood flow rate were set to Hct = 50% and *Q_b_* = 0.4 mL/h, respectively. (**i**) Timelapse *Q_mc_* and *Q_ac_* with respect to *C_dex_* = 5~20 mg/mL. (**ii**) Timelapse *μ_b_* and AI with respect to *C_dex_*. (**iii**) Variations in *μ_b_* and AI as a function of shear rate ($\dot{\gamma }$) with respect to *C_dex_*.

First, as shown in Figure 6A, blood viscosity (*μ_b_*) and RBC aggregation index (AI) were obtained with respect to *Q_b_* = 0.2~0.8 mL/h. Herein, test blood (Hct = 50%) was prepared by suspending normal RBCs into dextran solution (20 mg/mL). Figure 6(Ai) show timelapse *Q_mc_* and *Q_ac_* with respect to *Q_b_*. The corresponding *Q_mc_* of each *Q_b_* was obtained as *Q_mc_* = 0.2 ± 0.01 mL/h (*n* = 2042) for *Q_b_* = 0.2 mL/h, *Q_mc_* = 0.4 ± 0.03 mL/h (*n* = 1133) for *Q_b_* = 0.4 mL/h, *Q_mc_* = 0.6 ± 0.04 mL/h (*n* = 1101) for *Q_b_* = 0.6 mL/h, and *Q_mc_* = 0.8 ± 0.05 mL/h (*n* = 675) for *Q_b_* = 0.8 mL/h. Additionally, the corresponding *Q_ac_* of each *Q_b_* was obtained as *Q_ac_* = 0.01 ± 0.01 mL/h for *Q_b_* = 0.2 mL/h, *Q_ac_* = 0.03 ± 0.01 mL/h for *Q_b_* = 0.4 mL/h, *Q_ac_* = 0.04 ± 0.01 mL/h for *Q_b_* = 0.6 mL/h, and *Q_ac_* = 0.05 ± 0.01 mL/h for *Q_b_* = 0.8 mL/h. The results showed that both flow rates (*Q_mc_*, *Q_ac_*) remained stable over time and were accurately adjusted in proportion to *Q_b_*. Figure 6(Aii) shows timelapse *μ_b_* and AI with respect to *Q_b_*. The upper panel exhibits time-dependent *μ_b_* with respect to *Q_b_*. For the given *Q_b_* range, the shear rate in the main channel was estimated as $\dot{\gamma }$ = 133.3~533.3 s^−1^. Initially, *μ_b_* was almost independent of *Q_b_*. As time elapsed, its fluctuation increased significantly at lower *Q_b_* compared with higher *Q_b_*, owing to RBC sedimentation in the driving syringe rather than shear-thinning effect. The lower panel depicts timelapse AI with respect to *Q_b_*. As expected, lower *Q_b_* had a higher value of AI when compared with higher *Q_b_*. The AI tended to decrease significantly over time. As RBC sedimentation proceeded over time, the hematocrit of blood supplied into the microfluidic chip increased over time. For this reason, it was inferred that RBC sedimentation in a driving syringe contributed to increasing blood viscosity and AI continuously. As shown in Figure 6(Aiii), variations in *μ_b_* and AI were plotted as a function of shear rate ($\dot{\gamma }$). Lower *Q_b_* resulted in higher *μ_b_* and substantially higher AI compared with higher *Q_b_*. Given the strong influence of *Q_b_* on both parameters, *Q_b_* was fixed at 0.4 mL/h for the following experiments.

Second, as represented in Figure 6B, variations in blood viscosity (*μ_b_*) and RBC aggregation index (AI) were obtained as a function of the hematocrit. To probe the impact of the hematocrit on both properties, the hematocrit of the test blood was adjusted to Hct = 30%~60% by adding normal RBCs into dextran (20 mg/mL). The flow rate of the blood was fixed at *Q_b_* = 0.4 mL/h. As shown in Figure 6(Bi), timelapses *Q_mc_* and *Q_ac_* were obtained with respect to Hct. The *Q_mc_* remained nearly constant regardless of *Q_b_*, whereas *Q_ac_* tended to increase with respect to Hct. This increase was attributed to elevated junction pressure between the viscosity-sensing and aggregation-sensing channels, which resulted in increasing *Q_ac_* substantially. As shown in Figure 6(Bii), timelapses *μ_b_* and AI were obtained with respect to Hct. In the upper panel, the *μ_b_* initially increased with respect to the hematocrit and rose further over time. However, no marked difference was observed among Hct conditions. In the lower panel, the AI decreased significantly with increasing Hct and decreased continuously over time. As shown in Figure 6(Biii), variations in *μ_b_* and AI were plotted as a function of shear rate ($\dot{\gamma }$) with respect to Hct. Although *Q_b_* was kept constant, higher viscosity increased blood-filled width, which reduced shear rate in the viscosity-sensing channel. That is, higher viscosity led to a lower shear rate. The results showed that hematocrit contributed to increasing *μ_b_* significantly, whereas the AI decreased markedly with respect to Hct. Thus, aggregation index values should be interpreted with consideration of hematocrit-dependent effects. However, the AI did not exhibit substantial variations with respect to shear rate. The hematocrit of the test blood was then fixed at Hct = 50% in subsequent experiments.

Third, to investigate the impact of suspending medium on both properties, several different concentrations of dextran solution (*C_dex_* = 5~20 mg/mL) were used as blood medium. Herein, test blood (Hct = 50%) was prepared by adding normal RBCs into a specific dextran solution. Blood flow rate was set to *Q_b_* = 0.4 mL/h. As shown in Figure 6(Ci), timelapses *Q_mc_* and *Q_ac_* were obtained with respect to *C_dex_* = 5~20 mg/mL. According to the results, *Q_mc_* remained constant with respect to *C_dex_*. Additionally, the dextran solution did not contribute to varying *Q_ac_* substantially. Figure 6(Cii) exhibits timelapses *μ_b_* and AI with respect to *C_dex_*. With the exception of *C_dex_* = 5 mg/mL, dextran contributed to a time-dependent increase in blood viscosity. Blood suspended in *C_dex_* = 15 mg/mL exhibited the highest *μ_b_* and AI, while AI declined markedly over time. As shown in Figure 6(Ciii), variations in *μ_b_* and AI were plotted as a function of shear rate ($\dot{\gamma }$) with respect to *C_dex_*. The results showed that *μ_b_* changed markedly with shear rate. With the exception of *C_dex_* = 5 mg/mL, the shear-dependent variation in *μ_b_* was comparable among *C_dex_* = 10~20 mg/mL.

However, AI showed no substantial dependence on shear rate and exhibited large fluctuations, with the highest value observed at *C_dex_* = 15 mg/mL.

The experimental investigation demonstrated that syringe flow rate, hematocrit, and suspending medium had significant effects on blood viscosity and RBC aggregation index. Accordingly, to obtain consistent measurements, these three factors were strictly controlled in the following experiments.

### 3.5. Quantitative Comparison of RBC Aggregation Index Determined by Previous and Present Methods

In this subsection, the conventional method of RBC aggregation index (AI) was used to validate the AI obtained by the present method. According to the previous method, after blood flow was stopped, blood flow imaging was captured for 120 s. Timelapse image intensity was analyzed to get the RBC aggregation index [39,44]. Herein, to induce RBC aggregation under static conditions, the syringe pump was abruptly stopped after the blood flow had reached a stable state. Timelapse image intensity was acquired by analyzing blood images in the main channel (*I_mc_*). The contribution of hematocrit and dextran concentration to AI was determined by previous and proposed methods.

**Figure 7:**
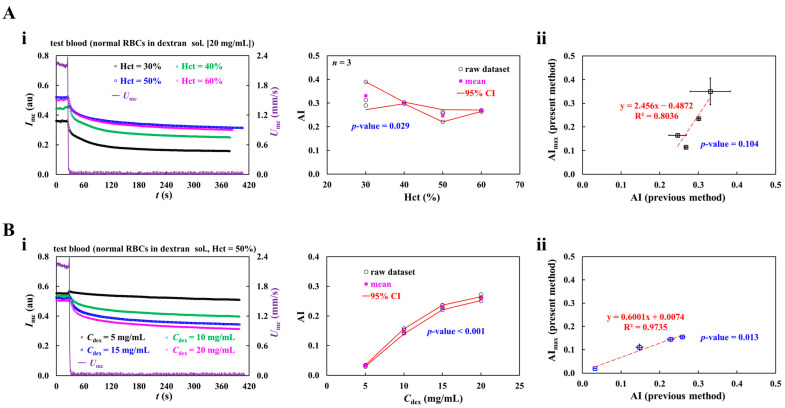
Quantitative comparison of RBC aggregation index (AI) measured by previous and present methods. (**A**) Contribution of hematocrit (Hct) to AI. (**i**) The effect of hematocrit on AI obtained by previous method. Herein, hematocrit of test blood was adjusted to Hct = 30%~60% by adding normal RBCs into dextran solution (20 mg/mL). Left-side panel shows time-course of I_mc_ and U_mc_ with respect to Hct. Right-side panel exhibits variations in AI with respect to Hct. (**ii**) Quantitative comparison of AI obtained by both methods as a function of hematocrit. Herein, AI (previous method) and AI_max_ (present method) denote AI obtained by the previous method and maximum AI obtained by the proposed method, respectively. (**B**) Contribution of dextran concentration to AI. (**i**) Effect of dextran concentration on AI determined using the previous method. Herein, test blood (Hct = 50%) was prepared by adding normal RBCs into dextran solution (*C*_dex_ = 5~20 mg/mL). Left-side panel shows timelapses of *I*_mc_ and *U*_mc_ with respect to C_dex_. Right-side panel depicts variations in AI with respect to *C*_dex_. (**ii**) Quantitative comparison of AI obtained by both methods as a function of dextran concentration.

First, to probe the contribution of hematocrit to RBC aggregation, the hematocrit of the test blood was adjusted to Hct = 30%~60% by adding normal RBCs into dextran solution (20 mg/mL). To stop blood flow immediately, polyethylene tubing was clamped with a pinch valve [64]. As shown in Figure 7(Ai), timelapses of *I*_mc_ and *U*_mc_ were obtained with respect to the hematocrit. After clamping, the *U*_mc_ was stopped without measurable delay, whereas the Imc gradually declined over time. In addition, the variation range of *I*_mc_ decreased as the hematocrit increased. The conventional RBC aggregation index (AI) was calculated by analyzing the time-course of *I*_mc_. As shown in the right-side panel of Figure 7(Ai), variations in AI were plotted as a function of the hematocrit. The red line denotes the 95% confidence interval (CI). The AI decreased as the hematocrit increased for up to 50%. However, no significant difference was observed between Hct = 50% and Hct = 60%. One-way ANOVA yielded a *p*-value of 0.029, which indicated that hematocrit significantly affected AI. The proposed method, unlike the previous one, measured AI during blood flow. RBC sedimentation in the driving syringe caused time-dependent hematocrit variation, which strongly influenced RBC aggregation. For convenience, the maximum AI obtained by the proposed method was adopted as the representative value. As shown in Figure 7(Aii), AI measured by the previous method and AI_max_ measured by the proposed method are plotted on the horizontal and vertical axes, respectively. Linear regression analysis showed a *p*-value = 0.104, indicating acceptable agreement between the RBC aggregation indices obtained by the two methods across hematocrit levels.

Second, to probe the effect of dextran concentration on the RBC aggregation index, test blood (Hct = 50%) was prepared by adding normal RBCs into dextran solution (*C*_dex_ = 5~20 mg/mL). As shown in the left-side panel of Figure 7(Bi), timelapses of *I*_mc_ and *U*_mc_ were acquired with respect to *C*_dex_. From the results, the variation range of *I*_mc_ increased markedly with increasing dextran concentration. Based on the conventional definition of the RBC aggregation index, the corresponding AI was determined for each dextran concentration. The right-side panel of Figure 7(Bi) exhibits variations in AI as a function of dextran concentration. One-way ANOVA yielded *p*-value < 0.0001, confirming that dextran concentration significantly increased AI. For comparison with the previous method, as shown in Figure 6C, the maximum AI was calculated as a function of dextran concentration. As shown in Figure 7(Bii), maximum AI determined by the proposed method and AI obtained by the conventional method are plotted on horizontal and vertical axes, respectively. Linear regression analysis gave *p*-value = 0.013, suggesting that the RBC aggregation indices obtained by the two methods were comparable across dextran concentrations.

According to quantitative comparison, the aggregation index responded sensitively to hematocrit and dextran concentration and showed good agreement with the conventional method. Thus, the present method was regarded as effective for evaluating RBC aggregation in microfluidic blood flow.

### 3.6. Evaluation of No-Delivery Waiting Time Under RBC Sedimentation

In Section 3.2, RBC sedimentation during continuous blood delivery with a syringe pump strongly affected blood viscosity and the RBC aggregation index. In this section, no-delivery waiting time (*T_w_*), which was defined as the elapsed time after stopping the syringe pump, was introduced as a new factor to accelerate RBC sedimentation. Test blood (Hct = 50%) was prepared by suspending normal RBCs in dextran solution (20 mg/mL). The corresponding flow rate of blood and 1× PBS was set to *Q_b_* = 0.4 mL/h and *Q_r_* = 1.8 mL/h, respectively.

**Figure 8:**
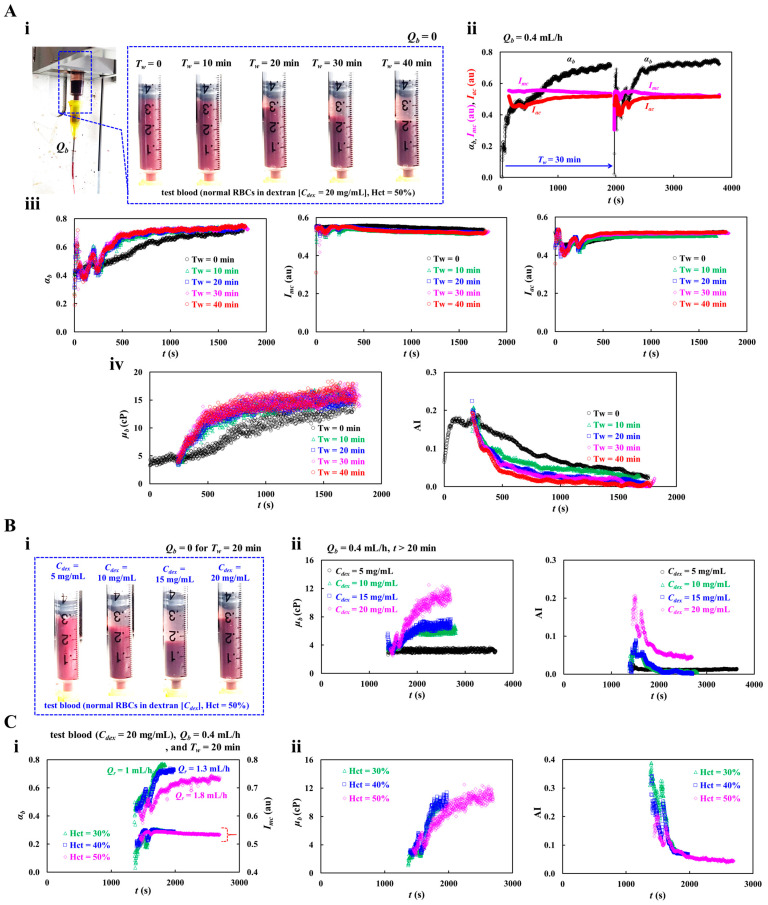
Quantitative evaluation of RBC sedimentation in a driving syringe using blood viscosity and RBC aggregation index. (**A**) Impact of no-delivery waiting time (*T_w_*) to blood viscosity (*μ_b_*) and RBC aggregation index (AI). Herein, test blood (Hct = 50%) was prepared by suspending normal RBCs into dextran solution (20 mg/mL). Flow rate of blood was fixed at *Q_b_* = 0.4 mL/h. (**i**) Snapshots showing RBC sedimentation with respect to no-delivery waiting time (*T_w_* = 0~40 min). (**ii**) Timelapses *α_b_*, *I_mc_*, and *I_ac_* with respect to *T_w_* = 0, 30 min. Herein, flow rate of 1× PBS was set to *Q_r_* = 1.8 mL/h. (**iii**) Timelapses *α_b_*, *I_mc_*, and *I_ac_* with respect to *T_w_* = 0~40 min. (**iv**) Timelapses *μ_b_* and AI with respect to *T_w_*. (**B**) Contribution of dextran solution to RBC sedimentation in a driving syringe. Herein, test blood (Hct = 50%) was prepared by suspending normal RBCs into dextran solution (*C_dex_* = 5~20 mg/mL). (**i**) RBC sedimentation in a driving syringe as a function of *C_dex_* after no-delivery waiting time of *T_w_* = 20 min. (**ii**) Timelapse μ_b_ and AI with respect to C_dex_. After a no-delivery waiting time of 20 min, blood was supplied at *Q_b_* = 0.4 mL/h. (**C**) Contribution of hematocrit to RBC sedimentation in a driving syringe. No-delivery waiting time and blood flow rate were set to *T_w_* = 20 min and *Q_b_* = 0.4 mL/h. (**i**) Timelapse Imc and αb with respect to Hct. (**ii**) Timelapse *μ_b_* and AI with respect to Hct.

First, as shown in Figure 8A, the impact of no-delivery waiting time (*T_w_*) on blood viscosity (*μ_b_*) and RBC aggregation index (AI) was quantitatively evaluated. As depicted in Figure 8(Ai), to visualize RBC sedimentation in the driving syringe, snapshots were taken at specific no-delivery waiting times (*T_w_* = 0~40 min) under no blood flow conditions (*Q_b_* = 0). RBC sedimentation proceeded continuously and was clearly observed after an elapsed time of 20 min. Figure 4(Aii) exhibits timelapses *α_b_*, *I_mc_*, and *I_ac_* with respect to *T_w_* = 0, 30 min. At *T_w_* = 30 min, the rise time of *α_b_* became shorter, but its plateau value remained essentially unchanged. Additionally, the image intensity difference (Δ*I* = *I_mc_* − *I_ac_*) was reduced. The results indicated that no-delivery waiting time had a strong impact on *α_b_* and Δ*I*. To find out the contribution of *T_w_* to interface and image intensity, as shown in Figure 8(Aiii), timelapses *α_b_*, *I_mc_*, and *I_ac_* were acquired with respect to *T_w_* = 0~40 min. In the left panel, the time-course of *α_b_* exhibits a similar increasing trend for *T_w_* = 10~40 min, except for *T_w_* = 0. In an initial period of blood delivery, *α_b_* showed large fluctuations. During the no-delivery waiting period, RBC sedimentation occurred in the fluidic path, including the inlet tubing and syringe needle, resulting in a nonuniform hematocrit distribution. At Qb = 0.4 mL/h, the transit time from the needle tip to the inlet port was estimated to be 132 s. Therefore, after flow resumed, sufficient transit time and shear exposure were required to disperse RBC aggregates and restore a homogeneous RBC suspension before entry into the microfluidic chip [42,45,73]. The middle panel shows that *I_mc_* decreased significantly after *T_w_* = 10 min and then remained nearly independent of *T_w_*. In the right panel, *I_ac_* increased slightly after *T_w_* = 10 min. Figure 8(Aiv) shows timelapses *μ_b_* and AI with respect to *T_w_*. Compared with *T_w_* = 0, the rise time of *μ_b_* was markedly shortened at *T_w_* > 10 min, while its transient profile remained similar. In contrast, AI decreased substantially, and its variation depended on *T_w_*.

Second, at *T_w_* = 20 min, the contribution of dextran solution to RBC sedimentation in a driving syringe was evaluated by measuring time-course of blood viscosity and RBC aggregation index. Herein, test blood (Hct = 50%) was prepared by suspending normal RBCs into dextran solution (*C_dex_* = 5~20 mg/mL). As shown in Figure 8(Bi), snapshots illustrating RBC sedimentation in the driving syringe were acquired as a function of *C_dex_*. In general, higher dextran concentrations promoted faster RBC sedimentation [71,74]. Notably, however, sedimentation at *C_dex_* = 20 mg/mL was much lower than that at *C_dex_* = 15 mg/mL. After *T_w_* = 20 min, the flow rate of both fluids was set to *Q_b_* = 0.4 mL/h and *Q_r_* = 1.8 mL/h, respectively. Blood and 1× PBS were then supplied into each inlet of the microfluidic chip. Figure 8(Bii) exhibits timelapses *μ_b_* and AI with respect to *C_dex_*. At *C_dex_* = 5 mg/mL, both *μ_b_* and AI remained nearly constant over time, indicating negligible RBC sedimentation in the syringe. For *C_dex_* > 5 mg/mL, *μ_b_* increased markedly as dextran concentration increased. It showed a significant upward trend over time. AI increased with respect to *C_dex_*. However, it gradually decreased over time. Overall, the results demonstrated that RBC sedimentation strongly influenced both blood viscosity and AI.

Third, the contribution of the hematocrit to RBC sedimentation in a driving syringe was detected using time-course of blood viscosity and the RBC aggregation index. Herein, test blood was prepared by suspending normal RBCs into dextran solution. No-delivery waiting time and blood flow rate were set to *T_w_* = 20 min and *Q_b_* = 0.4 mL/h. Figure 8(Ci) shows timelapses I_mc_ and α_b_ with respect to Hct. Herein, the corresponding *Q_r_* of each hematocrit was set to *Q_r_* = 1 mL/h (Hct = 30%), *Q_r_* = 1.3 mL/h (Hct = 40%), and *Q_r_* = 1.8 mL/h (Hct = 50%). At Hct = 30%~40%, the rising time of *α_b_* was shortened when compared with Hct = 50%. The *I_mc_* did not show a substantial difference with respect to Hct. As shown in the left-side panel of Figure 8(Cii), timelapse variations in *μ_b_* were obtained with respect to Hct. The plateau level of *μ_b_* remained comparable over the tested hematocrit range and showed no appreciable difference between Hct = 30% and 40%. In contrast, the rise time of *μ_b_* was prolonged at Hct = 50%. As shown in the right-side panel of Figure 8(Cii), timelapse AI was acquired with respect to Hct. The results showed that AI was initially higher at low hematocrit. However, after a certain time, the hematocrit no longer had a significant effect on AI. In particular, at Hct = 30%~40%, RBC sedimentation proceeded quickly. Both *μ_b_* and AI did not exhibit substantial a difference with respect to Hct = 30% or 40%.

The experiments showed that no-delivery waiting time strongly affected the time-dependent changes in blood viscosity and RBC aggregation index. Longer waiting times accelerated RBC sedimentation in the driving syringe, thereby altering both parameters over time. The suspending medium also had a significant effect on blood viscosity and RBC aggregation index. Therefore, to ensure consistent measurements, blood was loaded without a waiting period (*T_w_* = 0).

### 3.7. Contribution of Blood Flow Condition in Quantification of Blood Properties

Previous studies commonly assessed the RBC aggregation index by abruptly stopping blood flow or shear [39,44,64,73,75]. In such on–off protocols, RBC aggregation was quantified under stationary blood, whereas blood viscosity was determined during the constant-flow phase [76]. Because blood exhibits time-dependent transient rheology, viscosity values obtained immediately after flow switching may be less reliable than those measured after the flow reached a stable plateau at a defined shear condition [47,52]. Therefore, the conventional on–off flow method may be suboptimal for the simultaneous measurement of blood viscosity and RBC aggregation. In this section, based on time-course of blood viscosity and RBC aggregation index, the present continuous-flow method was quantitatively compared with the previous on–off method. Herein, test blood (Hct = 50%) was prepared by suspending normal RBCs into dextran solution (20 mg/mL). The flow rate of each fluid was set to *Q_b_* = 0.4 mL/h and *Q_r_* = 1.8 mL/h. In the previous method, the flow was controlled with *T_on_*/*T_off_* values of 2/2, 4/4, and 8/8 min/min.

**Figure 9:**
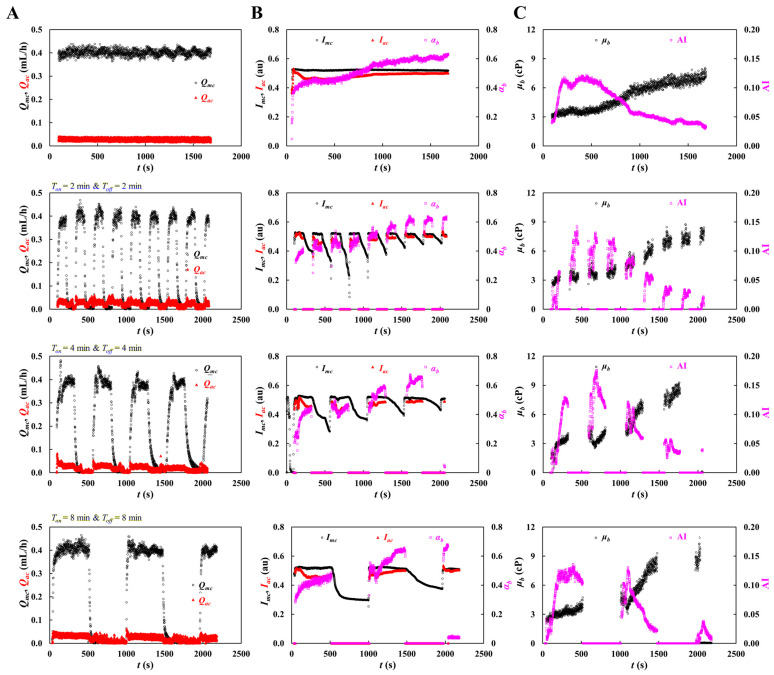
Quantitative comparison between present method (continuous blood flow) and previous method (on–off blood flow) using blood viscosity and RBC aggregation. Herein, test blood (Hct = 50%) was prepared by suspending normal RBCs into dextran solution (20 mg/mL). Flow rate of each fluid was set to *Q_b_* = 0.4 mL/h and *Q_r_* = 1.8 mL/h. For the previous method, flow rate of both fluids was set to *T_on_*/*T_off_* = 2/2, 4/4, and 8/8 min/min. (**A**) Timelapse *Q_mc_* and *Q_ac_* with respect to *T_on_*/*T_off_*. (**B**) Timelapse *I_mc_*, *I_ac_*, and *α_b_* with respect to *T_on_*/*T_off_*. (**C**) Timelapse *μ_b_* and AI with respect to *T_on_*/*T_off_*.

As shown in Figure 9A, timelapses *Q_mc_* and *Q_ac_* were obtained with respect to *T_on_*/*T_off_* = 0, 2/2, 4/4, and 8/8 min/min. The steady plateau value of *Q_mc_* was maintained at 0.4 mL/h. Under the constant flow rate condition, *Q_mc_* and *Q_ac_* remained stable over time. In contrast, under the on–off flow condition, shorter switching periods did not provide a sufficiently long steady plateau in *Q_mc_*. As the on–off period increased, the duration of the steady plateau also increased. Therefore, a longer on–off period, such as *T_on_*/*T_off_* = 8/8 min, was required to secure an adequately long plateau in the previous method. Figure 9B represents timelapses *I_mc_*, *I_ac_*, and *α_b_* with respect to *T_on_*/*T_off_*. According to the results, under continuous blood flow, the time-dependent profiles of *I_mc_*, *I_ac_*, and *α_b_* were obtained consistently and enabled appropriate analysis of blood changes. Under the on–off flow condition, however, *α_b_* did not show a consistent trend because it included transient behavior. In contrast, the temporal variations in *I_mc_* and *I_ac_* were similar to those observed under continuous blood flow. Figure 9C presents the time-dependent variations in *μ_b_* and AI with respect to *T_on_*/*T_off_*. The present method provided consistent temporal profiles of both parameters under continuous blood flow. Under the on–off flow condition, the overall profiles were similar to those obtained under continuous flow; however, because both *μ_b_* and AI included transient responses, the previous method had limitations in the quantitative analysis of their time-dependent variations.

The experimental results confirmed that the present method was superior to the previous one. In particular, accurate monitoring of blood changes requires continuous measurement of blood viscosity and RBC aggregation index under continuous blood flow.

### 3.8. Quantitative Evaluation of Heat-Exposed RBCs

In the last section, the present method was used to detect biophysical difference in thermally exposed RBCs. Previous studies have shown that exposure of RBCs to elevated temperature significantly alters hemorheological properties, including blood viscosity, RBC aggregation, and deformability [77,78,79,80]. Herein, normal RBCs were incubated at 40~50 °C for 20 min. Test blood (Hct = 50%) was then prepared by adding thermal-exposed RBCs into dextran solution (20 mg/mL). The corresponding flow rate of each was set to *Q_b_* = 0.4 mL/h and *Q_r_* = 1.8 mL/h.

**Figure 10:**
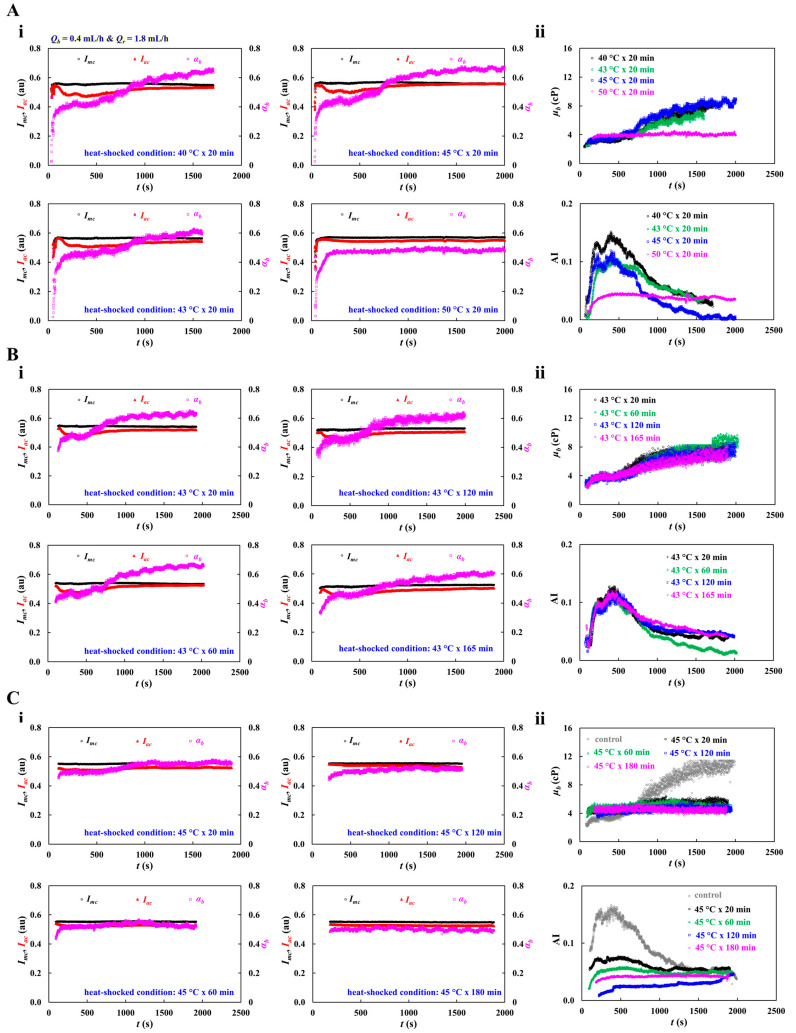
Quantitative evaluation of thermal-exposed RBCs using blood viscosity and RBC aggregation index. Herein, normal RBCs were incubated at 40~50 °C for 20 min. Test blood (Hct = 50%) was then prepared by adding thermal-exposed RBCs into dextran solution (20 mg/mL). The corresponding flow rate of each fluid was set to *Q_b_* = 0.4 mL/h and *Q_r_* = 1.8 mL/h. (**A**) Contribution of heat-exposed temperature to blood viscosity (*μ_b_*) and RBC aggregation index (AI). (**i**) Timelapses *I_mc_*, *I_ac_*, and *α_b_* with respect to heat-shocked condition (i.e., shock temperature = 40, 43, 45, and 50 °C, and exposed time = 20 min). (**ii**) Timelapses *μ_b_* and AI with respect to heat-exposed condition. (**B**) Contribution of exposed time to blood viscosity and RBC aggregation index. Herein, normal RBCs were incubated at the temperature of 43 °C. The exposed time was set to 20, 60, 120, and 165 min. (**i**) Timelapses *I_mc_*, *I_ac_*, and *α_b_* with respect to heat-exposed condition. (**ii**) Timelapses *μ_b_* and AI with respect to heat-exposed condition. (**C**) Influence of incubation time on blood viscosity and RBC aggregation index. Herein, incubation time was set to 20, 60, 120, and 180 min and exposed temperature was set to 45 °C. (**i**) Timelapses *I_mc_*, *I_ac_*, and *α_b_* with respect to heat-exposed time. (**ii**) Timelapses *μ_b_* and AI with respect to heat-exposed time.

First, the contribution of heat-exposure temperature to blood viscosity (*μ_b_*) and RBC aggregation index (AI) was quantitatively assessed. Figure 10(Ai) shows timelapses *I_mc_*, *I_ac_*, and *α_b_* with respect to heat-shock conditions (i.e., heat-exposure temperature = 40, 43, 45, and 50 °C, and exposed time = 20 min). Notably, *α_b_* showed little temporal variation under the high-temperature condition (50 °C for 20 min). Furthermore, the image intensity difference (Δ*I* = *I_mc_* − *I_ac_*) progressively decreased with increasing incubation temperature of normal RBCs. As shown in Figure 10(Aii), timelapses *μ_b_* and AI were obtained with respect to the heat-exposed condition. The upper panel exhibits timelapse *μ_b_* with respect to exposure temperature ranging from 40 °C to 50 °C. Initially, exposed time contributed to increasing *μ_b_* substantially. When normal RBCs were incubated at 50 °C, test blood remained nearly constant over time. After a certain time elapsed, the *μ_b_* was increased gradually over time. Timelapse *μ_b_* did show a slight difference with respect to exposed temperature. The lower panel presents the time-course of AI at different exposure temperatures. AI initially decreased substantially with increasing temperature. At 40~45 °C, it gradually decreased during blood delivery. However, it remained essentially unchanged at 50 °C. This indicated that RBC sedimentation was suppressed in the driving syringe after exposure to 50 °C, such that both *μ_b_* and AI remained constant during continuous blood loading.

Second, based on experimental results, to assess contribution of heat-exposure time to blood viscosity and RBC aggregation index, heat-exposure temperature was set to 43 °C. The exposed time increased from 20 min to 165 min. Figure 10(Bi) presents timelapses *I_mc_*, *I_ac_*, and *α_b_* with respect to the heat-exposed condition. Based on timelapses *I_mc_*, *I_ac_*, and *α_b_*, as shown in Figure 10(Bii), timelapses *μ_b_* and AI were obtained with respect to exposed time. According to the results, *μ_b_* increased gradually over time and showed no substantial dependence on exposure time. Similarly, the initial AI values did not differ markedly with exposure time. However, after a certain period, AI gradually decreased over time. Except for the 60 min exposure condition, the time-course of AI was independent of exposure time. Overall, incubation of normal RBCs at 43 °C for up to 120 min had no significant effect on the temporal variations in blood viscosity and RBC aggregation index.

Third, at an exposure temperature of 45 °C, incubation time increased from 20 min to 180 min. As shown in Figure 10(Ci), timelapses *I_mc_*, *I_ac_*, and *α_b_* were acquired with respect to heat-exposed time. The ΔI (Δ*I* = *I_mc_* − *I_ac_*) declined at longer exposure times, whereas *α_b_* was nearly constant over time except for the 20 min exposure. Figure 10(Cii) exhibits timelapses *μ_b_* and AI with respect to heat-exposed time. The upper panel presents time-course of *μ_b_* at different exposure times. Compared with the control, *μ_b_* of the test blood remained nearly constant over time and showed no substantial dependence on exposure time. The lower panel shows the time-course of AI at different exposure times. Compared with the control, AI of the test blood decreased markedly with increasing exposure time but remained nearly constant over time. These results suggest that after incubation at 45 °C for more than 20 min, blood viscosity and AI stayed stable because thermally shocked RBCs no longer contributed to sedimentation in the driving syringe. Thermal exposure time contributed to decreasing AI significantly.

The thermal treatment was used as a controlled experimental perturbation to alter RBC biophysical properties, rather than to directly replicate a specific clinical state. While 40~45 °C is associated with severe fever, hyperthermia, or local thermal exposure, 50 °C represents a supraphysiological condition used to probe pronounced RBC alteration. The reduced aggregation index could be interpreted as impaired reversible RBC aggregation caused by thermally altered membrane-mechanical properties. Because inflammation and diseases associated with reduced RBC deformability could modify aggregation behavior, these findings support the sensitivity of the proposed method to pathophysiological changes in RBC rheology [81,82].

The experimental results led to the conclusion that the proposed method could effectively detect hemorheological changes in thermal-shocked RBCs during continuous syringe delivery. Compared with control blood, heat-treated blood exhibited reduced AI and nearly constant time-course profiles of *μ_b_* and AI, indicating that thermal shock suppressed RBC sedimentation and aggregation. At higher temperatures and longer exposure times, these effects became more evident, suggesting that heat-induced RBC damage minimized their contribution to sedimentation in the syringe. Therefore, the proposed method could be regarded as useful for assessing heat-induced alterations in RBC function. As a limitation, experiments were performed at 25 °C to ensure stable and reproducible in vitro microfluidic measurements. As the correction factor used to improve blood viscosity was estimated from Newtonian fluid, its application to non-Newtonian blood may introduce systematic error. In the near future, the present method will be improved for conducting temperature-controlled measurements at 37 °C. In addition, as the present study focused on measuring effective blood viscosity in a microfluidic platform, it will be necessary to carry out detailed fitting with constitutive models (i.e., power-law model and Carreau–Yasuda model) by changing the blood flow rate, where RBC sedimentation does not have an influence on blood viscosity measurement. Furthermore, the present method will be employed to detect inter-donor variation in viscosity and RBC aggregation index.

## 4. Conclusions

This study proposed a microfluidic method for the simultaneous measurement of blood viscosity and RBC aggregation index under continuous blood delivery from a driving syringe. The results showed that both parameters were strongly affected by flow rate, hematocrit, suspension medium (dextran solution), and syringe on–off operation. In particular, flow interruption promoted RBC sedimentation in the syringe and consequently altered the measured hemorheological responses. These results highlighted the need to consider dynamic delivery conditions and demonstrated that the proposed method enabled reliable hemorheological analysis under continuous flow.

The method was further applied to characterize thermal-shock-induced changes in RBC behavior. Heat treatment reduced RBC aggregation and suppressed sedimentation, with these effects becoming more evident at higher temperatures and longer exposure times. Under severe thermal conditions, blood viscosity and aggregation index remained nearly constant over time, suggesting that thermally damaged RBCs no longer contributed substantially to sedimentation in the syringe. Therefore, the proposed method can serve as a sensitive and practical tool for simultaneous assessment of blood viscosity and RBC aggregation, as well as for detection of functional alterations in RBCs under continuous and non-interrupted blood flows. As a limitation, the proposed method was validated only with suspending blood. Thus, testing with patient blood samples will be required to verify its clinical applicability.

## Figures and Tables

**Table 1:** Comparison of previous studies related to simultaneous assessment of blood viscosity and RBC aggregation.

Platform Type	Parameters	Flow-Control	Ref.
Slit rheometer	RBC aggregation + viscosity with respect to shear rate	On–off pressure control	Shin et al. [50]
Microfluidic device	RBC aggregation size + viscosity with respect to shear rate	Low shearing blood flow	Mehri et al. [51]
Microfluidic closed fluidic circuit	RBC aggregation + viscosity	Period on–off blood flow supplied from a fluidic circuit	Kang [52]
Microfluidic device	RBC aggregation + viscosity	Stepwise varying flow rate control	Kang [41]
Microfluidic device	RBC aggregation + viscosity	Continuous blood flow	Present study

## Data Availability

The original contributions presented in this study are included in the article. Further inquiries can be directed to the corresponding author.
